# CTLA-4 Blockade Combined with PD-1/PD-L1 Inhibition Enhances Cytokine Production and T-Cell Activation in Ex Vivo Ovarian Cancer Cultures

**DOI:** 10.3390/ijms27125567

**Published:** 2026-06-19

**Authors:** Maitreyee Parulekar, Wook Ha Park, Miseon Kim, Kidong Kim, Jae Hong No, Yong Beom Kim, Dong Hoon Suh

**Affiliations:** Department of Obstetrics and Gynecology, Seoul National University Bundang Hospital, Seongnam 13620, Republic of Korea; mtry19@gmail.com (M.P.); summerwook@gmail.com (W.H.P.); shemme@naver.com (M.K.); kidong.kim.md@gmail.com (K.K.); jhno@snu.ac.kr (J.H.N.); ybkimlh@snubh.org (Y.B.K.)

**Keywords:** ovarian cancer, immunotherapy, programmed cell death 1 protein, T lymphocytes

## Abstract

Immune checkpoint inhibitors (ICIs) show promise in cancer but have limited efficacy in ovarian cancer. This study compared combinations of the PD-1/PD-L1 inhibitor with anti-LAG-3, anti-TIM-3, or anti-CTLA-4 to identify the most effective regimen by assessing T-cell CD8/CD4 ratios and cytokine production. T cells isolated from ovarian cancer tissues (mean 3.8 × 10^8^ cells) were stimulated and treated with the PD-1/PD-L1 inhibitor alone or combined with anti-LAG-3, anti-TIM-3, or anti-CTLA-4. Flow cytometry measured CD8/CD4 expression; ELISAs quantified TNF-α, IL-6, and IFN-γ. Anti-PD-1 monotherapy produced no significant change in CD8/CD4 ratio (1.36 ± 0.43 vs. 1.41 ± 0.36) or cytokine levels. Combination therapy with PD-1/PD-L1 inhibitor + anti-CTLA-4 induced the largest increase in CD8/CD4 ratio (3.69 ± 1.33, *p* < 0.001) compared with PD-1/PD-L1 inhibitor alone; increases were smaller for PD-1/PD-L1 inhibitor + anti-LAG-3 (2.11 ± 0.63, *p* = 0.009) and PD-1/PD-L1 inhibitor + anti-TIM-3 (1.87 ± 0.48, *p* = 0.026). TNF-α rose significantly only with PD-1/PD-L1 inhibitor + anti-CTLA-4 (106.69 ± 45.42 pg/mL, *p* = 0.008), not with PD-1/PD-L1 inhibitor + anti-LAG-3 (72.46 ± 31.79 pg/mL, *p* = 0.231) or PD-1/PD-L1 inhibitor + anti-TIM-3 (82.06 ± 33.63 pg/mL, *p* = 0.074). IFN-γ increase was greater with PD-1/PD-L1 inhibitor + anti-CTLA-4 than with PD-1/PD-L1 inhibitor + anti-LAG-3 (*p* = 0.026). In conclusion, dual PD-1/PD-L1 and CTLA-4 blockade induced concomitant increases in T-cell CD8/CD4 proportions and cytokine levels compared to monotherapy or alternative ICI pairings. These descriptive ex vivo observations offer preliminary evidence of altered immune profiles, highlighting this combination as a candidate for further functional validation.

## 1. Introduction

Antitumor immune responses are primarily attributed to cell-mediated immunity. Activation of CD4+ and CD8+ T cells is required for an effective immune response to destroy cancer cells [1]. Tumor infiltration with many CD8+ T cells is desirable and supports the role of tumor immune surveillance. The degree of tumor infiltration of intraepithelial CD8+ T cells is associated with improved survival in women with ovarian cancer [2,3]. This positive correlation provides a rationale for the use of immune checkpoint inhibitors (ICIs) in ovarian cancer [4]. For example, the programmed cell death protein-1 (PD-1) inhibitors target PD-1/programmed death-ligand 1 (PD-L1) interaction and consequently activate tumor-infiltrating CD8+ T cells to kill cancer cells [5]. Contrary to expectations, studies have shown that the efficacy of PD-1 pathway blockade in relapsed ovarian cancer remains limited, with a response rate of 10–15% [2]. Among the many factors related to this disappointing result was the low CD8+/CD4+ T-cell ratio, which indicates an immunosuppressive tumor microenvironment. CD4+CD25+FOXP3+ regulatory T cells (Tregs) in ovarian carcinoma suppress tumor-specific T-cell immunity and are associated with reduced survival [6]. Although CD8+/CD4+ T-cell ratios may differ in various types of cancer, this ratio is still considered a key indicator of appropriate tumor-infiltrating T-cell function [6,7,8].

Blockade of either the PD-1 receptor or its ligand PD-L1 has improved the overall survival in phase III clinical trials in patients with non-small-cell lung cancer, melanoma, and kidney cancer [9,10,11,12]. However, phase II trials of single-agent anti-PD-1/PD-L1 therapies in advanced recurrent ovarian cancer, including KEYNOTE-100 [13,14], NINJA [15], and JAVELIN [16], have yielded modest objective response rates (7.6–9.6%) and limited median progression-free survival (PFS) of 2.0–2.6 months. The modest single activity of various antibodies targeting PD-1/PD-L1 interaction in ovarian cancer suggests that a combination strategy may help overcome resistance to PD-1/PD-L1 blockade immunotherapy, given that co-inhibitory immune checkpoint molecules have been reported to increase in the tumor microenvironment and inhibit antitumor T-cell responses in most cancers [2,12,17].

Activated T cells have been known to express multiple co-inhibitory receptors, including PD-1, lymphocyte activation gene-3 (LAG-3), T-cell immunoglobulin and mucin domain-containing molecule-3 (TIM-3), B and T lymphocyte attenuator (BTLA), and CTLA-4 [18,19,20]. These co-inhibitory immune checkpoint molecules could be targeted as candidates for effective combination immunotherapy to restore antitumor immunity [21,22]. Among all immune checkpoint molecules, PD-1, LAG-3, TIM-3, and CTLA-4 have been widely studied [19,23]. Recently, a multidimensional immune profiling analysis revealed CTLA-4, LAG-3, and Treg as predictive for improved PFS in high-grade serous ovarian cancer [24]. Therefore, we selected LAG-3, TIM-3, and CTLA-4 as co-inhibitory targets for immunotherapy along with the PD-1/PD-L1 inhibitor.

In this pilot study, we utilized an exploratory ex vivo patient-derived mononuclear culture system to screen the associative effects of pairing a PD-1/PD-L1 inhibitor with additional checkpoint blockades (anti-CTLA-4, anti-LAG-3, or anti-TIM-3). By evaluating surrogate markers of immune profiles—specifically CD8/CD4 ratios and secreted effector cytokines—we aimed to map baseline phenotypic trends to guide future functional validation assays.

## 2. Results

### 2.1. Baseline Information

The baseline characteristics of the study population and the ovarian specimens are shown in Table 1. The mean age of the 10 patients with ovarian cancer was 51.9 ± 9.7 years. According to the 2018 International Federation of Gynecology and Obstetrics staging system, the ovarian specimens were classified as follows: four 1A, one 1C, two 3B, two 3C, and one IVB. The average tumor weight and isolated T-cell number retrieved from the study population were 3.91 g and 303.26 × 10^8^, respectively.

### 2.2. CD8/CD4 Ratio Before and After Different Combinations of Immunotherapy with PD-1/PD-L1 Inhibitor

**Figure 1:**
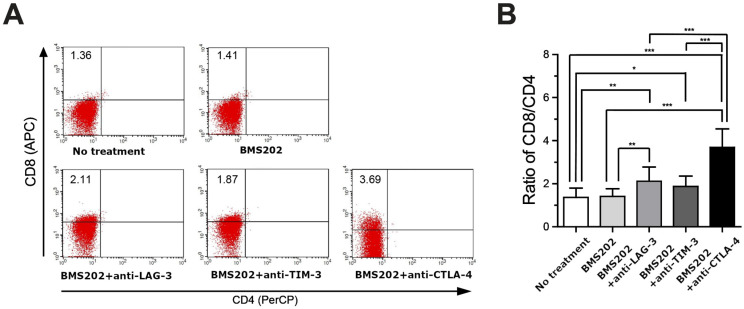
Ratio of CD8+/CD4+ T cells in ovarian cancer patient tumors. (**A**) Representative FACS plot of CD4+ and CD8+ of T lymphocytes in a patient tumor. The isolated T cells from ovarian cancer tissue were treated with BMS202 (PD-1/PD-L1 inhibitor), LAG-3, TIM-3, and CTLA-4 inhibitor for 48 h. (**B**) Quantification of CD8+/CD4+ ratio value in inhibitor-treated T-lymphocytes. Isolated T cells without treatment for 48 h were used as the control. Data are presented as the means ± S.D. (n = 10). Statistical significance across multiple groups was analyzed by one-way ANOVA followed by Tukey’s honestly significant difference (HSD) post hoc test (* *p* < 0.05, ** *p* < 0.01, *** *p* < 0.001 vs. no treatment group or between the indicated groups).

In the absence of treatment, the CD8/CD4 ratio in the isolated T cells was 1.36 ± 0.43 in 10 ovarian cancer tissues (Figure 1). This ratio was almost equal to that of normal ovarian tissue (1.40). After treatment with PD-1/PD-L1 inhibitor alone, the CD8/CD4 ratio in T cells increased slightly, but the difference was not statistically significant (1.41 ± 0.36, *p* = 0.678).

Compared to PD-1/PD-L1 inhibitor monotherapy, all dual combinations significantly elevated the CD8/CD4 ratio: anti-LAG-3 (2.11 ± 0.63, *p* = 0.009), anti-TIM-3 (1.87 ± 0.48, *p* = 0.026), and anti-CTLA-4 (3.69 ± 1.33, *p* < 0.001). Statistically significant differences were observed in T cells treated with PD-1/PD-L1 inhibitor + anti-LAG-3 (*p* = 0.009), PD-1/PD-L1 inhibitor + anti-TIM-3 (*p* = 0.026), and PD-1/PD-L1 inhibitor + anti-CTLA-4 (*p* < 0.001) antibodies compared with those treated with PD-1/PD-L1 inhibitor alone. Among the three combined strategies, PD-1/PD-L1 inhibitor + anti-CTLA-4 antibodies had the highest CD8/CD4 ratio compared with PD-1/PD-L1 inhibitor + anti-LAG-3 and PD-1/PD-L1 inhibitor + anti-TIM-3 (*p* < 0.001 for both).

### 2.3. Cytokine Levels Before and After Immunotherapy

**Figure 2:**
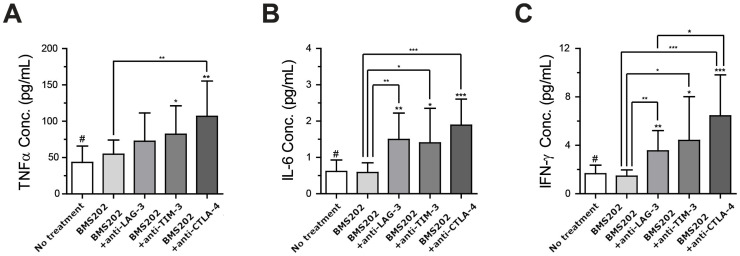
The induction of cytokines in the T cells isolated from ovarian cancer tissue. T cells were untreated or were treated with BMS202 (PD-1/PD-L1 inhibitor), anti-LAG-3, anti-TIM-3, or anti-CTLA-4 antibodies for 48 h. The production of (**A**) TNFα, (**B**) IL-6, and (**C**) IFN-γ was then detected in the cell culture supernatant using ELISA assay kits. Data are presented as the means ± S.D. (n = 10). Statistical significance across multiple groups was analyzed by one-way ANOVA followed by Tukey’s HSD post hoc test (* *p* < 0.05, ** *p* < 0.01, *** *p* < 0.001 vs. # no treatment group or between the indicated groups).

Without any treatment, TNF-α, IL-6, and IFN-γ levels were determined in 10 ovarian cancer tissues as 43.19 ± 22.68, 0.61 ± 0.26, and 1.65 ± 0.71 pg/mL, respectively (Figure 2).

After treatment with PD-1/PD-L1 inhibitor alone, the levels of TNF-α (54.61 ± 19.44 pg/mL, *p* = 0.109), IL-6 (0.58 ± 0.27 pg/mL, *p* = 0.102), and IFN-γ (1.45 ± 0.52 pg/mL, *p* = 0.534) did not show any significant differences.

Compared with T cells not subjected to any treatment (marks of statistical significance are not shown in Figure 2), treatment with PD-1/PD-L1 inhibitor + anti-LAG-3 antibodies increased TNF-α (72.46 ± 34.46 pg/mL, *p* = 0.054), IL-6 (1.49 ± 0.71 pg/mL, *p* = 0.002), and IFN-γ (3.55 ± 1.58 pg/mL, *p* = 0.004), while treatment with PD-1/PD-L1 inhibitor + anti-TIM-3 antibodies significantly increased the levels of all cytokines (TNF-α: 82.06 ± 36.95 pg/mL, *p* = 0.014; IL-6: 1.40 ± 0.79 pg/mL, *p* = 0.023; IFN-γ: 4.40 ± 2.90 pg/mL, *p* = 0.030). Treatment with PD-1/PD-L1 inhibitor + anti-CTLA-4 antibodies demonstrated a concomitant increase in the levels of TNF-α (106.69 ± 49.23 pg/mL, *p* = 0.001), IL-6 (1.89 ± 0.85 pg/mL, *p* < 0.001), and IFN-γ (6.43 ± 3.42 pg/mL, *p* < 0.001).

Compared with T cells treated with PD-1/PD-L1 inhibitor alone (marks of statistical significance are shown in Figure 2), TNF-α was significantly increased only in the T cells treated with PD-1/PD-L1 inhibitor + anti-CTLA-4 antibodies (106.69 ± 45.42 pg/mL, *p* = 0.008). However, no significant increase of TNF-α was observed in the T cells treated with PD-1/PD-L1 inhibitor +anti-LAG-3 (72.46 ± 31.79 pg/mL, *p* = 0.231) or PD-1/PD-L1 inhibitor + anti-TIM-3 (82.06 ± 33.63 pg/mL, *p* = 0.074). By contrast, the levels of IL-6 and IFN-γ increased in all the T-cell groups treated with three combinations of antibodies (IL-6: PD-1/PD-L1 inhibitor + anti-LAG-3, 1.49 ± 0.72 pg/mL, *p* = 0.002; PD-1/PD-L1 inhibitor + anti-TIM-3, 1.04 ± 0.81 pg/mL, *p* = 0.023; PD-1/PD-L1 inhibitor + anti-CTLA-4, 1.89 ± 0.86 pg/mL, *p* < 0.001 and IFN-γ: PD-1/PD-L1 inhibitor + anti-LAG-3, 3.56 ± 1.64 pg/mL, *p* = 0.002; PD-1/PD-L1 inhibitor + anti-TIM-3, 4.40 ± 2.99 pg/mL, *p* = 0.027; PD-1/PD-L1 inhibitor + anti-CTLA-4, 6.43 ± 3.52 pg/mL, *p* = 0.0004). In addition, IFN-γ increase was greater in the T cells treated with PD-1/PD-L1 inhibitor + anti-CTLA-4 than those treated with PD-1/PD-L1 inhibitor + anti-LAG-3 (*p* = 0.026).

### 2.4. Immunohistochemistry Staining

**Figure 3:**
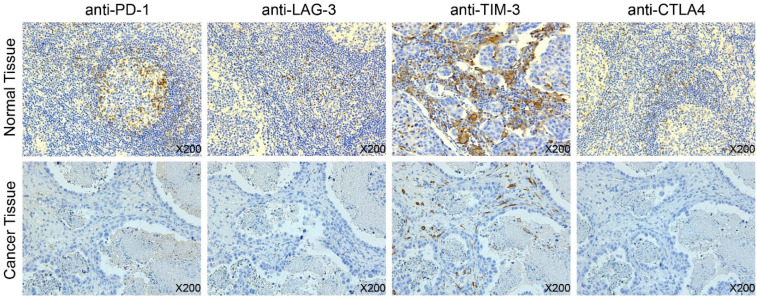
Representative immunohistochemical staining for PD-1, LAG-3, TIM-3, and CTLA-4 expression in ovarian cancer tissue. The expressions of PD-1 (blue-gray), LAG-3 (brown), TIM-3 (brown), and CTLA-4 (blue-gray) were detected in normal or ovarian cancer tissue. Resected tumor tissues were immunostained with antibody panel containing 3 recombinant rabbit monoclonal antibodies against human LAG-3, TIM-3, and CTLA-4 and 1 mouse monoclonal antibody against human PD-1. Original magnification: ×200.

The heterogeneous expression of PD-1, LAG-3, TIM-3, and CTLA-4 receptors in normal ovarian tissue and ovarian cancer tissues is shown in Figure 3. No consistent pattern was observed in the expression of PD-1, LAG-3, TIM-3, or CTLA-4 receptors in ovarian cancer tissues.

## 3. Discussion

PD-L1 expression in tumor cells is common in various cancers, including lung (50%), esophagus (44%), stomach (42%), kidney (37%), breast (34%), and ovarian (68.6%) [25]. PD-L1 expression in tumor cells is considered more prevalent in ovarian cancer than in other cancers and may play a crucial role in suppressing host–tumor immunity against highly immunogenic tumors. Hamanishi et al. reported that PD-L1 expression in tumor cells and the number of intraepithelial infiltrating CD8+ T cells are independent prognostic factors in women with ovarian cancer [25]. They showed a significant negative correlation between PD-L1 expression and the number of intraepithelial CD8+ T lymphocytes, suggesting that PD-L1 on tumor cells inhibits anti-tumor CD8+ T cells. Immunotherapy, particularly ICIs, has emerged as a promising approach for treating ovarian cancer, although results with single-agent ICI have been modest. In line with clinical non-responsiveness to monotherapy, single-agent PD-1/PD-L1 blockade failed to alter either the CD8/CD4 ratio or any evaluated effector cytokine levels. However, our exploratory ex vivo profiling indicates that while single-agent PD-1/PD-L1 inhibition yields minimal changes, pairing blockades shifts the local immune profile. Among the tested pairs, the dual PD-1/CTLA-4 combination demonstrated the most pronounced shifts in immune profiles, elevating both the CD8/CD4 ratios and core effector cytokines. These findings offer preliminary hypothesis-generating evidence. While these co-occurring phenotypic changes suggest altered immune profiles, further independent functional validation —such as direct cytotoxicity or tumor-killing assays—is mandatory to establish a definitive anti-tumor mechanism. While large-scale clinical trials evaluating dual blockades of nivolumab (PD-1 inhibitor) and tremelimumab (anti-CTLA-4) have noted divergent outcomes—ranging from therapeutic signal expansions [17,26] to limited activity in heavily pretreated cohorts [27]—our ex vivo observations serve strictly as a preliminary ex vivo tissue profile rather than a predictive model for clinical therapeutic outcomes.

The rationale for evaluating anti-CTLA-4, anti-TIM-3, and anti-LAG-3 alongside PD-1 inhibition stems from the known heterogeneity and compensatory upregulation of these pathways within the ovarian tumor microenvironment [28,29]. Prior literature indicates that dual blockades, particularly PD-1 and CTLA-4 co-targeting, can restore T-cell proliferation and cytokine release by overcoming compensatory resistance mechanisms [30,31,32]. Our observation that alternative combinations (such as anti-TIM-3 or anti-LAG-3 pairs) yielded less pronounced shifts than anti-CTLA-4 further reflects the complex, multi-layered nature of adaptive immune resistance, which is often constrained by alternative checkpoint networks or immunosuppressive cell types like Tregs [23,33,34,35]. This multi-layered complexity is further supported by our baseline immunohistochemistry findings, which revealed highly heterogeneous checkpoint receptor expression profiles with no consistent pattern across patient samples. This distinct initial receptor patchiness likely underscores the wide intra-group variability observed in our ex vivo combination assays, reflecting a major translational challenge for biomarker-driven cohorts.

This study provides valuable insights into the potential of combination immunotherapy for ovarian cancer treatment. Key strengths include the direct comparison of different antibody combinations in ovarian cancer tissue samples and the simultaneous assessment of multiple immune response markers. These findings offer a phenotypic rationale for the potential trends observed in clinical dual-blockade settings. However, several limitations are present. Due to the small cohort size (n = 10) and the inherent heterogeneity of tumor stages and histological subtypes in our samples, substantial data variability and borderline *p*-values were observed across treatment groups. Consequently, these baseline descriptive findings constrain the statistical power and generalizability of our study, and do not allow for a definitive, clinically predictive ranking of the tested ICI combinations. Another limitation is the absence of CD3-based quantification to formally confirm T-cell enrichment in the Percoll intermediate fraction. To address this constraint, future work will integrate CD3/CD4/CD8 immunophenotyping on newly collected samples. Additionally, the intentional use of LPS to activate residual antigen-presenting cells (APCs) within our mononuclear cultures introduces a baseline inflammatory confounder. The resulting cytokine surges must therefore be interpreted with caution, as they represent a hybrid response driven by both immune checkpoint blockade and global TLR4-mediated APC activation rather than isolated T-cell receptor pathways. Furthermore, the ex vivo nature of these cultures may not fully recapitulate the dynamic tumor microenvironment in vivo. Because we did not perform direct cytotoxicity assays, our results must be interpreted strictly as surrogate markers of immune activation rather than definitive proof of a superior antitumor response. Additionally, this investigation did not explore potential biomarkers that could predict patient response to these combination therapies. Further validation using larger cohorts, in vivo models, patient-derived organoids, or advanced cellular vectors (such as genetically engineered TCR or CAR T cells) remains necessary to validate and expand upon these preliminary findings.

## 4. Materials and Methods

### 4.1. Ovarian Tissue Collection

Ten patients with ovarian cancer scheduled for surgery were prospectively recruited at the Seoul National University Bundang Hospital, Republic of Korea, between June 2017 and December 2019 (Clinical trial number: not applicable). Tumor samples were specifically collected from ovarian masses. One patient scheduled for benign ovarian tumor resection was recruited as the control, which was used as a baseline reference for immunohistochemistry and initial ratio comparisons. This study was conducted in accordance with the Declaration of Helsinki and was approved by the Institutional Review Board of Seoul National University Bundang Hospital (approval no. B-1708/412-302). Written informed consent to participate and to publish their clinical details and/or clinical images was obtained from all the patients prior to the collection of tissue samples.

### 4.2. Reagents and Antibodies

Anti-CD4 (BD#347324) and -CD8 (BD#340584) antibodies were obtained from BD Biosciences (Franklin Lakes, NJ, USA), and lipopolysaccharide (LPS) from *Escherichia coli* O111:B4 was purchased from Sigma-Aldrich (St. Louis, MO, USA). BMS202 (PD-1/PD-L1 inhibitor, a small-molecule inhibitor of the PD-1/PD-L1 interaction), relatlimab (LAG-3 inhibitor), sabatolimab (TIM-3 inhibitor), and MDK 24720 (CTLA-4 inhibitor) were purchased from Selleckchem (Houston, TX, USA). Human high-sensitivity enzyme-linked immunosorbent assay (ELISA) kits for IFN-γ, IL-6, and TNF-α were purchased from Thermo Fisher Scientific (Waltham, MA, USA).

### 4.3. T-Cell Isolation, Purification, and Staining

To analyze lymphocyte activity, tumor specimens were digested with 1 mg/mL collagenase type IV (Worthington Biochemicals, Lakewood, NJ, USA) and 1 mg/mL DNase I (Sigma-Aldrich, St. Louis, MO, USA) for 30 min at 37 °C with gentle mixing. During the final 5 min of incubation, a Percoll density gradient (Sigma-Aldrich, St. Louis, MO, USA) was applied. After enzymatic digestion and Percoll density separation, the collected mononuclear fraction (enriched for T cells) was used for downstream culture and analyses. We did not perform CD3 staining to quantify T-cell purity in this cohort; instead, downstream phenotyping and functional assays were performed on the Percoll-enriched mononuclear fraction, and CD8/CD4 proportions were determined by flow cytometry, as reported in Figure 1. The Percoll intermediate fraction corresponds to the PBMC-like (mononuclear) fraction in standard density separation protocols (see manufacturer’s instructions at https://www.sigmaaldrich.com/KR/en/technical-documents/protocol/cell-culture-and-cell-culture-analysis/mammalian-cell-culture/how-to-make-and-use-gradients-of-percoll?srsltid=AfmBOoo9K_zXaOAnCIMmoSHwXP5ouFsK746WSVsLpi7W9cf_y6HxC4De) (accessed on 1 January 2026).

For single-cell analysis, the collected mononuclear fraction enriched for T cells was plated and maintained in complete medium (10% FBS, 1% penicillin–streptomycin) with 1 μg/mL phytohemagglutinin for 48 h in a T-75 flask to stimulate and expand lymphocytes prior to staining. Cells were then centrifuged, resuspended, and stained with anti-CD8 (1:200) and anti-CD4 (1:400) antibodies (BD Biosciences, Franklin Lakes, NJ, USA) for 1 h at room temperature before analysis by flow cytometry (BD FACS Calibur, v5.1, BD Biosciences, San Jose, CA, USA).

### 4.4. Cell Culture and Treatment

The mononuclear fraction enriched for T cells was resuspended in RPMI 1640 medium containing 10% heat-inactivated FBS and β-mercaptoethanol (Sigma-Aldrich, St. Louis, MO, USA). To provide co-stimulatory signals for secondary T-cell activation, μg/mL LPS (*E. coli* O111:B4) was added to activate residual monocytes/APCs within the mononuclear fraction. The cells were then treated with the PD-1/PD-L1 inhibitor BMS202 (10 nM), relatlimab (LAG-3 inhibitor, 10 μg/mL), sabatolimab (TIM-3 inhibitor, 20 μg/mL), or MDK 24720 (CTLA-4 inhibitor, 20 nM), as indicated. Treated cells were incubated at 37 °C in a 5% CO2 atmosphere for 48 h prior to flow cytometry and supernatant collection.

### 4.5. Flow Cytometry

Anti-CD4 (PerCP, 1:400) or anti-CD8 (APC, 1:200) antibodies were used for flow cytometry analysis. Cultured cells were collected 48 h after stimulation and/or inhibitor treatment, washed, stained, and analyzed on a FACS Calibur. Representative gating and analysis for CD8/CD4 used for downstream analyses are shown in Figure 1.

### 4.6. Enzyme-Linked Immunosorbent Assay (ELISA) for Cytokines

The culture supernatants of the treated T cells were removed 48 h after the different treatments and kept at 80 °C until cytokine measurement. TNF-α, IL-6, and IFN-γ levels were selected as cytokines that reflect the T-cell effector functions associated with PD-1 expression. This was determined using a commercially available high-sensitivity ELISA kit (Thermo Fisher Scientific; Bender MedSystems, Vienna, Austria) in 96-well microtiter plates according to the manufacturer’s instructions. Absorbance was measured on a spectrophotometer at 450 nm using a SpectraMax M Series (M5) Microplate Reader (Molecular Devices, San Jose, CA, USA). The concentration of cytokines in each sample was obtained by extrapolating a standard calibration curve generated simultaneously. The cytokine levels were quantified in pg/mL.

### 4.7. Immunohistochemistry

Formalin-fixed paraffin-embedded sections of human ovarian tumor and non-tumor tissues were used for immunohistochemical staining. These tissues were fixed in 5% paraformaldehyde solution, dehydrated, and embedded in paraffin. For microscopy analysis (×200, Olympus BX53M, Tokyo, Japan), 5 mm paraffin-embedded tissue sections were stained with anti-PD-1 (20 μg), anti-LAG-3 (20 μL), anti-TIM-3 (20 μL), anti-CTLA-4 (20 μL), and anti-TIGIT (20 μL) (Abcam, Cambridge, UK). TIGIT was used as a T-cell exhaustion marker. All the slides were examined by at least one pathologist in a blinded manner. Slide images were prepared using Adobe Photoshop and Illustrator CS3 (Adobe Systems Inc., San Jose, CA, USA).

### 4.8. Statistics

Data are presented as mean ± standard deviation. Comparisons between two groups were performed using two-tailed unpaired Student’s *t*-tests. For multiple group comparisons, one-way analysis of variance (ANOVA) was performed, followed by Tukey’s honestly significant difference (HSD) post hoc test to control for the family-wise error rate. Statistical analyses were performed using Prism (GraphPad) software (version 5.0; GraphPad Software Inc., San Diego, CA, USA). Statistical significance was set at *p* < 0.05.

## 5. Conclusions

Our findings offer preclinical insights into the potential synergistic effects of combination immunotherapy involving the PD-1/PD-L1 inhibitor and other ICIs in ovarian cancer. Further clinical investigations are necessary to validate these results.

## Figures and Tables

**Table 1:** Baseline characteristics of the study population and ovarian specimens used for T-cell isolation.

Number	Age (Year)	FIGO Stage	CA-125 (U/mL)	Histologic Type	Number of Isolated T Cells (×10^8^)
Control	71	NA	117.6	Brenner tumor & mucinous cystadenoma	492.81
1	50	3C	>1000	HGS	324.17
2	40	3B	1020	HGS	256.24
3	47	1A	32.4	Endometrioid	41.28
4	48	1A	33.5	Mucinous	48.26
5	46	1C	317.4	Clear cell	147.31
6	55	3C	4110.4	HGS	384.62
7	38	3B	179.6	HGS	391.45
8	59	1A	62.7	Clear cell	408.74
9	56	1A	11.0	Clear cell	374.59
10	61	IVB	983.8	Carcinosarcoma (HGS 95% + UD sarcoma 5%)	466.38

FIGO, International Federation of Gynecology and Obstetrics; CA-125, cancer antigen-125; NA, not applicable; HGS, high-grade serous; UD, undifferentiated.

## Data Availability

The original contributions presented in this study are included in the article. Further inquiries can be directed to the corresponding author.
